# Cathepsin B Improves ß-Amyloidosis and Learning and Memory in Models of Alzheimer’s Disease

**DOI:** 10.1007/s11481-016-9721-6

**Published:** 2016-12-13

**Authors:** Christine M Embury, Bhagyalaxmi Dyavarshetty, Yaman Lu, Jayme L Wiederin, Pawel Ciborowski, Howard E Gendelman, Tomomi Kiyota

**Affiliations:** 1 Department of Pharmacology and Experimental Neuroscience, University of Nebraska Medical Center, Omaha, NE USA; 2 Department of Internal Medicine, University of Nebraska Medical Center, 985880 Nebraska Medical Center, Omaha, NE 68198-5880 USA

**Keywords:** Adeno-associated virus, Gene therapy, Lysosomal degrading enzyme, Proteomics, Radial arm water maze

## Abstract

Amyloid-ß (Aß) precursor protein (APP) metabolism engages neuronal endolysosomal pathways for Aß processing and secretion. In Alzheimer’s disease (AD), dysregulation of APP leads to excess Aß and neuronal dysfunction; suggesting that neuronal APP/Aß trafficking can be targeted for therapeutic gain. Cathepsin B (CatB) is a lysosomal cysteine protease that can lower Aß levels. However, whether CatB-modulation of Aß improves learning and memory function deficits in AD is not known. To this end, progenitor neurons were infected with recombinant adenovirus expressing CatB and recovered cell lysates subjected to proteomic analyses. The results demonstrated Lamp1 deregulation and linkages between CatB and the neuronal phagosome network. Hippocampal injections of adeno-associated virus expressing CatB reduced Aß levels, increased Lamp1 and improved learning and memory. The findings were associated with the emergence of c-fos + cells. The results support the idea that CatB can speed Aß metabolism through lysosomal pathways and as such reduce AD-associated memory deficits.

**Electronic supplementary material:**

The online version of this article (doi:10.1007/s11481-016-9721-6) contains supplementary material, which is available to authorized users.

## Introduction

Alzheimer’s disease (AD) is the most common form of cognitive impairment affecting the elderly (Selkoe 1991). There is neither a cure nor an effective therapy for slowing this progressive neurodegenerative disorder or in targeting the neuropathological disease hallmarks. Prominent characteristics of the disease include the formation of extracellular amyloid-ß (Aß) plaques, intraneuronal Aß accumulation and the neurofibrillary tangles known to cause neuronal death and linked memory impairments (Mattson 2004; Billings et al. 2005; Oakley et al. 2006). Developing these accumulations begins with Aß precursor protein (APP) processing. APP is internalized and sorted within endosomes, where APP is processed to generate Aß. Aß is subsequently degraded by the endolysosomal pathway, or released into the extracellular space by fusion of multivesicular bodies with the plasma membrane. This is but one operative mechanism for Aß-mediated AD pathogenesis that is linked to the production of exosomes, beginning the aggregation process (Rajendran et al. 2006; Rajendran and Annaert 2012; Pacheco-Quinto and Eckman 2013). In the AD aged brains, chronic inflammation contributes to aberrant Aß clearance and aggregation (Krstic and Knuesel 2013). Alterations in the endolysosomal/autophagosomal pathways, responsible for APP/Aß trafficking and degradation, are likely a driving mechanism involved in this protein dysregulation process (Rajendran and Annaert 2012). Dysregulation of these pathways can accelerate AD pathology. This occurs through a buildup of nondegraded proteins, leakage of lysosomal contents and apoptosis (Tung et al. 2012). APP processing can occur within accumulated compartments with more Aß42 species produced compared to less toxic forms of the Aß protein (Tung et al. 2012). In all, endolysomal/autophagosomal dysfunctions result in intraneuronal Aß accumulation that leads to synaptic dysfunction and cognitive impairment (Oddo et al. 2003; Knobloch et al. 2007). These all suggest that targeting neuronal APP/Aß trafficking underlies neuronal survival in the diseased brain and may improve clinical outcomes.

Cathepsin B (CatB), a lysosomal cysteine protease, mediates proteolysis within lysosomes (Alvarez et al. 2012). In general, it plays a number of roles in phagocytosis and autophagy, growth/tumor cell proliferation, angiogenesis, invasion, and metastasis (Mort and Buttle 1997; Aggarwal and Sloane 2014). In regard to AD, abundant CatB immunoreactivity is operative extracellular to and within neuronal perikarya. It is also associated within senile plaques as seen in the postmortem AD brains (Cataldo et al. 1990). CatB present in the plaques is extracellular to lysosomal dense bodies and lipofuscin granules. Plaque formation is likely a result of lysosomal protease-mediated APP processing present in degenerating neurons (Cataldo and Nixon 1990). In support of such observations are studies seeking to attenuate CatB expression in AD. These experiments were performed in animal models demonstrating that pharmacological or gene CatB inhibition results in reduced ß-amyloidosis and improvements in memory function (Hook et al. 2007; Hook et al. 2008; Hook et al. 2009; Hook et al. 2011; Kindy et al. 2012). Not withstanding, recent studies reported conflicting results. In these studies CatB was found less expressed in patients with dementia and CatB deletion by its inhibitors increased Aß levels (Wang et al. 2012; Tiribuzi et al. 2014). In such works, overexpression of CatB lowered Aß levels (Mueller-Steiner et al. 2006; Yang et al. 2011; Wang et al. 2012). As CatB is involved in c-terminal truncation and as such Aß clearance (Mueller-Steiner et al. 2006; Butler et al. 2011) the findings suggests that it plays a beneficial role in AD progression. With such conflicting results, it is imperative that the mechanisms behind Aß clearance and the role played by CatB overexpression on learning and memory function are unraveled.

Thus, we sought to directly address the effects of CatB on Aß production. Our approach was to use recombinant adenovirus (Ad) expressing HA-tagged human CatB (Ad-CatB) to unravel CatB-mediated protein modulation. These studies were completed in neural progenitor cell (NPC)-derived neurons to ensure homogenous selective cell cultures. To further explore mechanisms we employed proteomics assays to validate cell-based influences of neuronal function. Concurrently, adeno-associated virus (AAV) serotype 2/1 recombinant expressing the CatB (AAV-CatB) was employed to uncover “putative” learning and memory deficits as seen in an AD animal model of human disease. APP/presenilin-1 (PS1) double-transgenic (Tg) mice (APP/PS1 mice) received intracranial AAV injections with subsequent studies of behavioral, neuropathological, and biochemical analyses. The data demonstrated clear associations between CatB and lowered levels of amyloidogenesis and improvements in learning and behavioral functions. The results lay the groundwork for new therapeutic testing and developments in a disease with few treatment options.

## Materials and Methods

### Neural Progenitor Cell (NPC) Cultivation

NPCs were prepared using the NeuroCult Proliferation Kit (StemCell Technologies, Vancouver, BC, Canada) according to manufacturer’s instruction. In brief, mouse cortices were dissected at embryonic day 14 and meninges were removed in ice-cold PBS with 2% glucose. The cortices were mechanically dissociated, filtered with a 40 μm-cell strainer and cultured as neurospheres for 3–5 days in NeuroCult Proliferation media with epidermal growth factor (20 ng/ml). The neurospheres were collected and dissociated to single cells using a NeuroCult Chemical Dissociation Kit (StemCell Technologies, Vancouver, BC, Canada). The cells were seeded into poly-D-lysine (100 μg/ml) and laminin (15 μg/ml; Sigma-Aldrich, St. Louis, MO)-coated tissue culture plates. Proliferation media were exchanged with Neurobasal media containing B-27 supplement and 1 x penicillin/streptomycin for differentiation.

### Recombinant Adenovirus Generation and Infections

Recombinant Ad expressing cystatin B (AdCysB) and AdCatB (both co-expressing GFP) were generated using AdEasy™ XL Adenoviral Vector System (#240010), pShuttle-IRES-hrGFP-1 vector (#240081) and pShuttle-IRES-hrGFP-2 vector (#240082, all from Agilent Technologies, Santa Clara, CA, USA) according to manufacturer’s instruction. A PCR fragment containing CysB was amplified using the primers: Fw: 5′- TACGATTTAGGTGACACTATAG -3′ (SP6), Rev.: 5′-TTTCCTCGAGGAAATAGGTCAGCTCATC -3′ and pCMV-SPORT6 containing human CysB coding sequence (Open Biosystems clone 2900656) as a template DNA, digested with *Eco* RV and *Xho* I and inserted into the multiple cloning site (MCS) of pShuttle-IRES-hrGFP-1. A PCR fragment containing CatB was amplified using the primers: Fw: 5′- GGATCTAGGATCCGGCTTCCAAC -3′, Rev.: 5′- GATCCTCGAGGATCTTTTCCCAGTACTG -3′ and pCMV-SPORT6 containing human CatB coding sequence (Open Biosystems clone 30334082) as a template DNA, digested with *Bam* HI and *Xho* I and inserted into the MCS of pShuttle-IRES-hrGFP-2 to generate pShuttle-CatBHA-IRES-hrGFP-2. Recombinant AdGFP and AdAPPsw (co-expressing GFP) were generated as previously described (Kiyota et al. 2015b). Viral titer was measured using AdEasy™ Viral Titer Kit (#972500, Agilent Technologies, Santa Clara, CA, USA). Differentiated NPCs were infected with adenoviruses (MOI = 10 per each) in 200 μl fresh Opti-MEM (Life Technologies, Carlsbad, CA, USA) for 1 h, followed by washing with PBS and 1-day incubation in Neurobasal media. The media were subjected to Aß40 or Aß42 ELISA (Life Technologies, Carlsbad, CA, USA) according to manufacturer’s instruction. Cell lysates were prepared and subjected to immunoblot and proteomic analyses.

### Quantitative Proteomics by SWATH-MS

NPCs were seeded at a density of 4.0 × 10^5^ per well in a 24-well plate and differentiated with neurobasal media for 24 h prior to further treatment. Cells were infected with AdGFP or AdCatB as described above. At the 72-h post infection time point, cells were washed 3 times with ice-cold PBS, then lysed on ice with 100 μl of 2% (*w*/*v*) SDS in 100 mM Tris-HCL, pH 7.6, supplemented with protease and phosphatase inhibitors (final concentration 1×; Thermo Fisher Scientific, Waltham, MA, USA) per well. Lysate was collected in 1.7 ml sterile microcentrifuge tubes, pipetting repeatedly to further breakdown cellular components, kept on ice. 0.5 μl benzonase (Millipore, Darmstadt, Germany) was added per tube to breakdown DNA and to make the lysate solution less viscous. Lysate was thoroughly mixed by pipet and vortexed shortly before being spun down at 10,000 g for 10 min at 4 °C to pellet and remove cellular debris. Supernatant was transferred into a new sterile 1.7 ml tube, flash frozen on dry ice, and stored at −80 °C until proceeding with proteomic analysis.

Protein concentration was determined using a BCA kit (Thermo Fisher Scientific, Waltham, MA, USA) following the manufacturer’s instructions. Prior to digestion, DTT was added to cell lysates for a final concentration of 25 mM and samples were heated at 95 °C for 5 min. Samples were allowed to cool and were processed as previously described (Haverland et al. 2014; Arainga et al. 2015) using filter aided sample preparation (FASP) digestion of 50 μg per sample. Following overnight digestion, samples were cleaned using Oasis MCX column (Waters Corporation, Milford, MA, USA), followed by C18 Zip-Tips (Thermo Fisher Scientific). Cleaned peptides were quantitated using NanoDrop2000 by A205.

Following resuspension, 1 μg of sample was used for SWATH-MS analysis as previously described (Haverland et al. 2014). Briefly, 1μg of each sample was injected through Eksigent cHiPLC column (75 μm × 15 cm ChromXP C18-CL 3 μm 120 Å) onto 5600 TripleTOF (Sciex) using a typical gradient 2–60% ACN in 60 min. Experimental samples used for SWATH were spiked with HRM calibration peptides (Biognosys AG) for retention time correction during data analysis and data was acquired using Data-Independent Acquisition. Samples for the library were acquired using Data Dependent Acquisition (DDA) and all files searched together using ProteinPilot 4.2 Uniprot_SwissProt database (2014) for one output. Protein Pilot identified 1242 proteins at 1% FDR Global Fit confidence and these proteins were imported into PeakView (Sciex) software for SWATH processing.

Raw data was transformed by natural log (ln) and z-scores, z-test and z-*p* values were computed per treatment condition, as previously described (Cheadle et al. 2003; Haverland et al. 2014). Z-test and associated *p*-values were computed using a standard normal distribution. Bioinformatics tools were used to discern relationships between proteins found significantly different (*p* < 0.05), specifically between AdCatB and AdGFP, as well as AdCatB and control conditions. UniProt IDs were entered into the Protein Analysis Through Evolutionary Relationships (PANTHER) classification system and the Kyoto Encyclopedia of Genes and Genomes (KEGG) pathway analysis to determine major pathways affected by treatment. The proteins changed within an identified major pathway were colored pink and cyan for up- and down-regulation respectively indicating positively identified proteins.

### AAV Generation

A PCR fragment containing 3× HA was amplified using the primers: Fw: 5′- CTCACGGGGATTTCCAAGTC -3′, Rev.: 5′- GCCTAAGCTTAGGCGTAGTCAGGTACAT -3′ and pShuttle-IRES-hrGFP-2 as a template DNA, digested with *Nhe* I and *Hind* III and inserted into the MCS of pAAV2-CBA-MCS-WPRE (AAV2 inverted terminal repeats flanking cytomegalovirus immediate early enhancer, chicken ß-actin promoter with first exon and intron sequences, MCS, Woodchuck hepatitis post-transcriptional regulatory element, and the bovine growth hormone polyadenylation site (Kiyota et al. 2011) to construct pAAV2-CBA-MCS-HA-WPRE. To construct pAAV2-CatB, a PCR fragment containing CatB as described above was digested with *Bam* HI and *Xho* I and inserted into the MCS of pAAV2-CBA-MCS-HA-WPRE. AAV-GFP was generated using a pGFP vector (Klein et al. 2002; Kiyota et al. 2010). AAV-293 cells (#240073, Agilent Technologies, Santa Clara, CA, USA) were co-transfected with *cis* plasmid pAAV2-CatB or pGFP, an AAV1 *trans* plasmid p5E18RXC1 and a helper plasmid pAd∆F6 (obtained from University of Pennsylvania Gene Therapy Program) to produce AAVs. Cells were harvested, AAVs were purified and titration performed (Kiyota et al. 2009, 2011 #31).

### AAV Transduction

Differentiated NPCs seeded at a density of 400,000 cells (24-well) were transduced with AAVs in 200 μl Neurobasal media, then 300 μl fresh media were added 24 h after transduction. Cells were harvested using ice-cold RIPA buffer (Thermo Fisher Scientific, Waltham, MA, USA) with protease inhibitor cocktail (Sigma, St. Louis, MO, USA) 3 days after AAV transduction. Protein concentrations were determined using a Micro BCA Protein Assay (Thermo Fisher Scientific, Waltham, MA, USA).

### 3-(4,5-Dimethylthiazol-2-Yl)-2,5-Diphenyltetrazolium Bromide (MTT) Assay

Differentiated NPCs were seeded at a density of 50,000 cells (96-well) were transduced with indicated amounts of AAVs in 50 μl Neurobasal media for 24 h, then 50 μl fresh media were added. Three days after transduction cells were incubated with 10 μl of MTT (*ATCC*® 30-1010 K) for 4 h at 37 °C. Media were aspirated from each well and 50 μl of dimethyl sulfoxide was added to dissolve the formazan crystals, and absorbance was measured using a plate reader at 570 nm.

### Transgenic Mice

APP/PS1 double-transgenic mice were bred as previously described (Kiyota et al. 2011). Age-matched non-Tg mice in B6/129 F1 strain (Jackson laboratory, Bar Harbor, ME, USA) were maintained by intercrossing in the same facility. All animal work performed in this study adhered to the guidelines established by the Institutional Animal Care and Use Committee at University of Nebraska Medical Center.

### Stereotaxic Injection

Mice at 3 months of age received *i.p.* injection of ketamine/xylazine anesthesia (100 mg/kg ketamine and 20 mg/kg xylazine). After mice were immobilized in a stereotaxic microinjection frame (Stoelting, Wood Dale, IL, USA), a linear skin incision was made exposing the bregma, and a 1-mm burr hole was drilled in the skull 2.1 mm posterior and 1.8 mm lateral to the bregma on both sides using a hand-held driller (Craftsman). A total volume of 2 μl of saline containing AAVs (1 × 10^9^ vg) was injected into hippocampus using Hamilton syringe (Hamilton, Reno, NV, USA) equipped with a 30-gauge needle at 0.2 μl/min at a depth of 1.8 mm below the skull.

### Tissue Preparation

Four months post-injection, mice were deeply euthanized with isoflurane and transcardially perfused with 25 ml of ice-cold PBS, followed by 4% PFA/PBS (Sigma-Aldrich). The brains were rapidly removed. The left hemisphere was dissected and immediately frozen in dry ice for biochemical testing. The right hemisphere was immersed in freshly depolymerized 4% paraformaldehyde for 48 h at 4 °C, and protected by successive 24-h immersions in 15% and 30% sucrose in 1 x PBS. Fixed, cryopreserved brains were sectioned coronally using a Cryostat (Leica, Bannockburn, IL, USA) with sections serially collected and stored at −80 °C for immunohistochemical tests. For biochemical testing, protein extraction of an extracellular-enriched fraction was extracted as described (Lesne et al. 2006). After separation of extracellular-enriched fraction, protein pellet was homogenized in ice-cold RIPA buffer (Thermo Fisher Scientific, Waltham, MA, USA) with protease inhibitor cocktail (Sigma, St. Louis, MO, USA). Protein concentration was determined using Micro BCA Protein Assay (Thermo Fisher Scientific, Waltham, MA, USA).

### Immunoblots

Protein lysates were diluted 1:1 with Laemmli buffer containing ß-mercaptoethanol, incubated at 100 °C for 5 min, electrophoresed on 10% SDS-polyacrylamide tris-tricine gels or tris-glycine gels, and electroblotted to 0.45-μm pore size PVDF membranes (Immobilon-P, Millipore, Billerica, MA, USA). Membranes were blocked in 5% skim milk/TBST, and incubated with Aß monoclonal (6E10, 1:1000, Covance, Emeryville, CA, USA), GFP rabbit polyclonal (1:5000, Abcam, Cambridge, MA, USA), Flag monoclonal (M2, 1:5000, Sigma, St. Louis, MO, USA), HA monoclonal (HA-7, 1:5000, Sigma, St. Louis, MO, USA) or Lamp1 rabbit polyclonal (1:1000, ab24170, Abcam, Cambridge, MA, USA) at 4 °C for overnight, followed by 30-min incubation in 5% skim milk/TBST with HRP-conjugated anti-mouse or rabbit IgG antibodies (Ab) (1: 2000, Santa Cruz Biotechnology, Santa Cruz, CA, USA). Immunoreactive bands were detected with SuperSignal West Pico or Femto Chemiluminescent substrate (Thermo Fisher Scientific, Waltham, MA, USA) and captured using a FluorChem M MultiFluor system (ProteinSimple, Santa Clara, CA, USA) or a myECL Imager (Thermo Fisher Scientific, Waltham, MA, USA). After detection of the bands, membranes were incubated with Restore Western Blot Stripping Buffer (Thermo Fisher Scientific, Waltham, MA, USA) and were then used to detect ß-actin for normalization using HRP-conjugated anti-ß-actin monoclonal (1: 5000, Sigma, St. Louis, MO, USA). For quantitative analysis, ImageJ software (NIH, Bethesda, MD, USA) was used to quantify band intensities relative to control on captured images.

### Immunofluorescence

Immunofluorescence was performed using specific Abs to Lamp1 (1:1000, ab24170, Abcam, Cambridge, MA, USA) and HA (HA-7, 1:5000, Sigma, St. Louis, MO, USA). Alexa Fluor 488 goat anti-mouse IgG and Alexa Fluor 568 goat anti-rabbit IgG (Life Technologies, Carlsbad, CA, USA) were used as secondary. Images were captured using a 63X oil lens on a LSM 710 confocal microscope (Carl Zeiss Microimaging Inc., Thornwood, NY, USA). Images were quantified as occupied areas per cell bodies using ImageJ software (NIH, Bethesda, MD, USA).

### Immunohistochemistry

Immunohistochemistry was performed as described (Kiyota et al. 2011) using specific Abs to identify glial fibrillary acidic protein (GFAP, rabbit polyclonal, 1:2000, DAKO, Carpenteria, CA, USA), ionized calcium binding adaptor molecule 1 (Iba1, rabbit polyclonal, 1:1000, Wako, Richmond, VA, USA), pan-Aß (rabbit polyclonal, 1:100, Zymed, San Francisco, CA, USA) and c-fos (rabbit polyclonal, 1:5000, Calbiochem, Gibbstown, NJ, USA). Immunodetection was visualized using biotin-conjugated anti-rabbit IgG was used as a secondary Ab, followed by a tertiary incubation with Vectastain ABC Elite kit (Vector Laboratories, Burlingame, CA, USA). The areas of Aß loads were analyzed by ImageJ software (NIH, Bethesda, MD, USA) at 300 μm intervals in twelve 30 μm coronal sections from each mouse. Seven mouse brains per group were analyzed.

### Stereological Quantification

We defined cells with nuclei that were DAB stained in the granular cell layer (GCL) of the dentate gyrus (DG) as c-fos ^+^ cells (Kiyota et al. 2015a). Positive cells were counted in a blinded fashion in every 8th section through the entire anterio–posterior extent of the DG (total 12 sections per hippocampus) and estimated using stereological analysis with Stereo Investigator system with an optical fractionator module (MBF Bioscicence, Williston, VT). The system consisted of a high sensitivity digital camera (OrcaFlash2.8, Hamamatsu C11440-10C, Hamamatsu, Japan) interfaced with a Nikon Eclipse 90i microscope (Nikon, Melville, NY, USA). Within the Stereo Investigator program, the contour of DG of each section was delineated using a tracing function. While sections showed shrinkage along the anterio–posterior axis, the extent of shrinkage between different animals was similar. The dimensions for the counting frame (450 × 450 um) and the grid size (500 × 500 um) were set. The z-plane focus was adjusted at each section for clarity, and images were automatically acquired according to each setting. The data file containing all slice pictures were quantified by the fractionator and marked positive cells in the analyzed areas of the DG that were observed in each counting frame. Based on these parameters and marked cell counts, the Stereo Investigator program computed the estimated cell population. These total markers, cell counts and the Gunderson (m = 1) values were recorded for each animal and compared between groups using a statistical software (Prism 4.0, Graphpad Software, San Diego, CA).

### Radial Arm Water Maze Test

The radial arm water maze (RAWM) task was run as described (Kiyota et al. 2013). Animals were introduced into the perimeter of a circular water-filled tank (21–22 °C) 110 cm in diameter and 91 cm in height (San Diego Instruments, San Diego, CA) with triangular inserts placed in the tank to produce six swim paths radiating from a central area. Spatial cues for mouse orientation were present on the tank walls. At the end of one arm, a 10 cm circular plexiglass platform was submerged 1 cm deep and as such hidden from the mice. The platform was located in the same arm for four consecutive acquisition trials (T1 through T4), and one 30-min delayed retention trial (T5), but in a different arm on different days. For T1-T4, the mouse started the task from a different randomly chosen arm, excluding the arm with the platform. After four trials, the mouse was returned to its cage for 30 min, and then administered the retention trial (T5) starting from the same arm as in T4. Each trial lasted 1 min and an error was scored each time when the mouse, excluding tail, entered the wrong arm, entered the arm with the platform but did not climb on it, or did not make a choice for 20 s. The trial ended when the mouse climbed onto and remained on the hidden platform for 10 s. The mouse was given 20 s to rest on the platform between trials. After each trial, mice were gently wiped using paper towels to remove aqueous droplet then put back into cages on a warming pad. The errors over 9-day test were divided into three blocks, and the errors in each block consisting of 3-day test were averaged for statistical analysis.

### Statistics

All data were normally distributed and presented as mean values ± standard errors of the mean (SEM). In case of single mean comparison, data were analyzed by Student’s *t*-test. In case of multiple mean comparisons, the data were analyzed by one-way ANOVA and Newman-Keuls *post-hoc* or two-way repeated measures ANOVA, followed by Bonferroni multiple comparison tests using statistics software (Prism 4.0, Graphpad Software, San Diego, CA). A value of *p* < 0.05 was regarded as significant.

## Results

### CatB Suppresses NPC-Derived Neuronal Aß Production

**Fig. 1:**
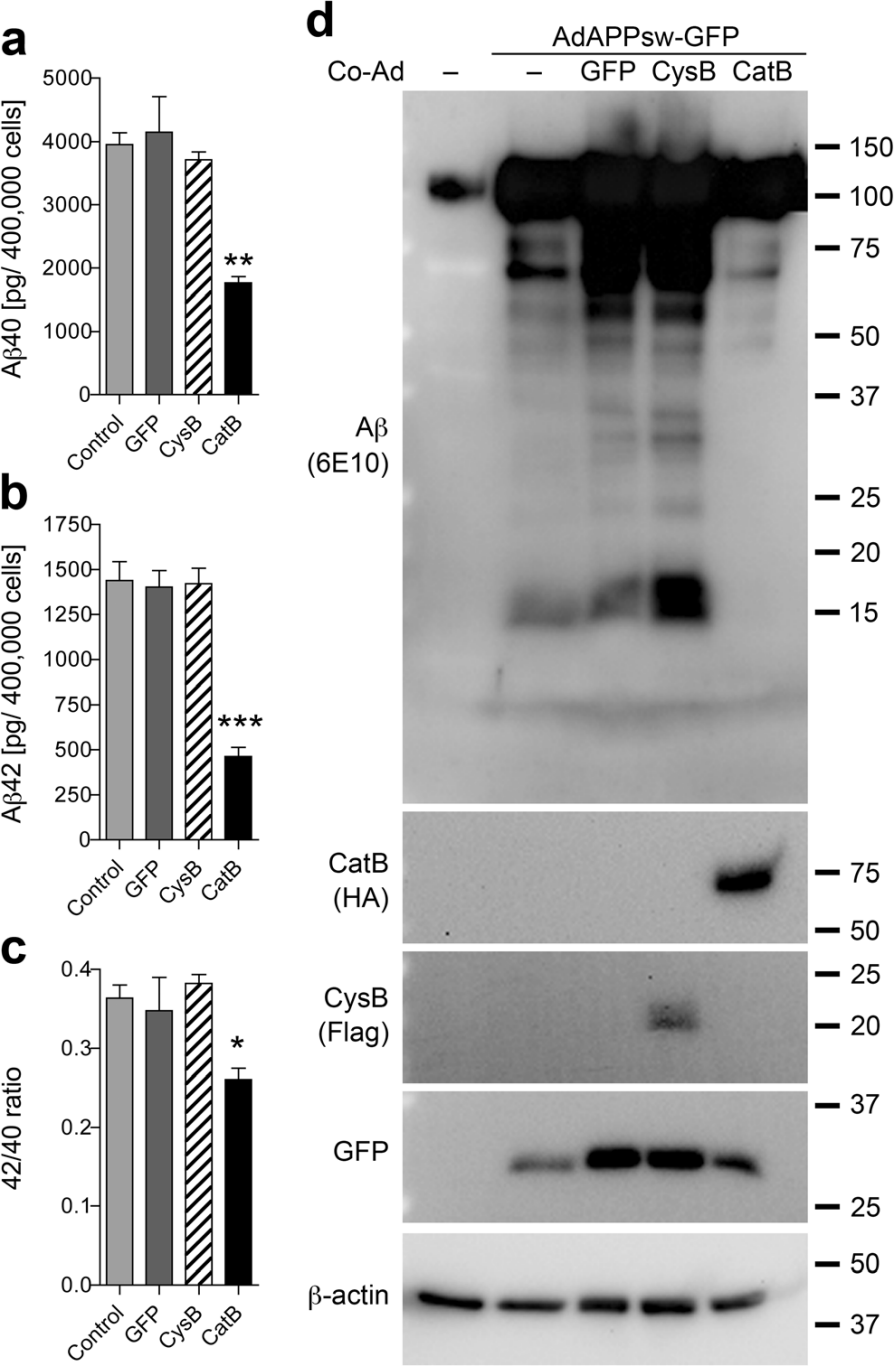
CatB suppresses Aß production from mouse NPC-derived neurons. **a, b** NPC-derived neurons were infected with AdAPPsw or co-infected with AdAPPsw and AdGFP, AdCysB or AdCatB. Aß40 (a) or Aß42 (b) production was quantified, and Aß42/Aß40 ration **c** was calculated. **d** Aß retention in neurons was examined using 6E10 antibody (top). Expression of CatB (HA-tagged CatB), CysB (Flag-tagged CysB) and GFP were to validate experimental condition (bottom). Bars represent mean ± S.E.M. *p < 0.05, **p < 0.01, ***p < 0.001, one-way ANOVA, Newman-Keuls post hoc test

CatB is known to decrease Aß concentrations by cleavage of Aß into smaller fragments (Mueller-Steiner et al. 2006). To confirm and extend these results CatB-mediated modulation of Aß was investigated in NPC-derived neurons infected with Ad expressing GFP (AdGFP), human APP Swedish mutant (AdAPPsw) or co-infected with AdAPPsw and AdGFP, AdCatB or Ad expressing Flag-tagged cystatin B (AdCysB: an endogenous CatB inhibitor and a positive control for Aß aggregation (Ceru et al. 2010; Smajlovic et al. 2011). Conditioned media were subjected to Aß40 or Aß42-specific ELISA, showing that CatB significantly reduced production of Aß40 (44.6% of control, Fig. 1a) and Aß42 (32.1% of control, Fig. 1b) from the neurons, and Aß42/Aß40 ration (71.6% of control, Fig. 1c). To examine Aß retention in neurons, cell lysates were subjected to immunoblot assays using Aß antibody (Fig. 1d). CysB enhanced Aß protein expression as seen by increased intensity band through a wide range of molecular weights, suggesting it promoted aggregation. Opposingly, CatB facilitated Aß degradation and inhibited its production in neurons.

### Proteomics Analyses of AdCatB-Infected Neurons

**Fig. 2:**
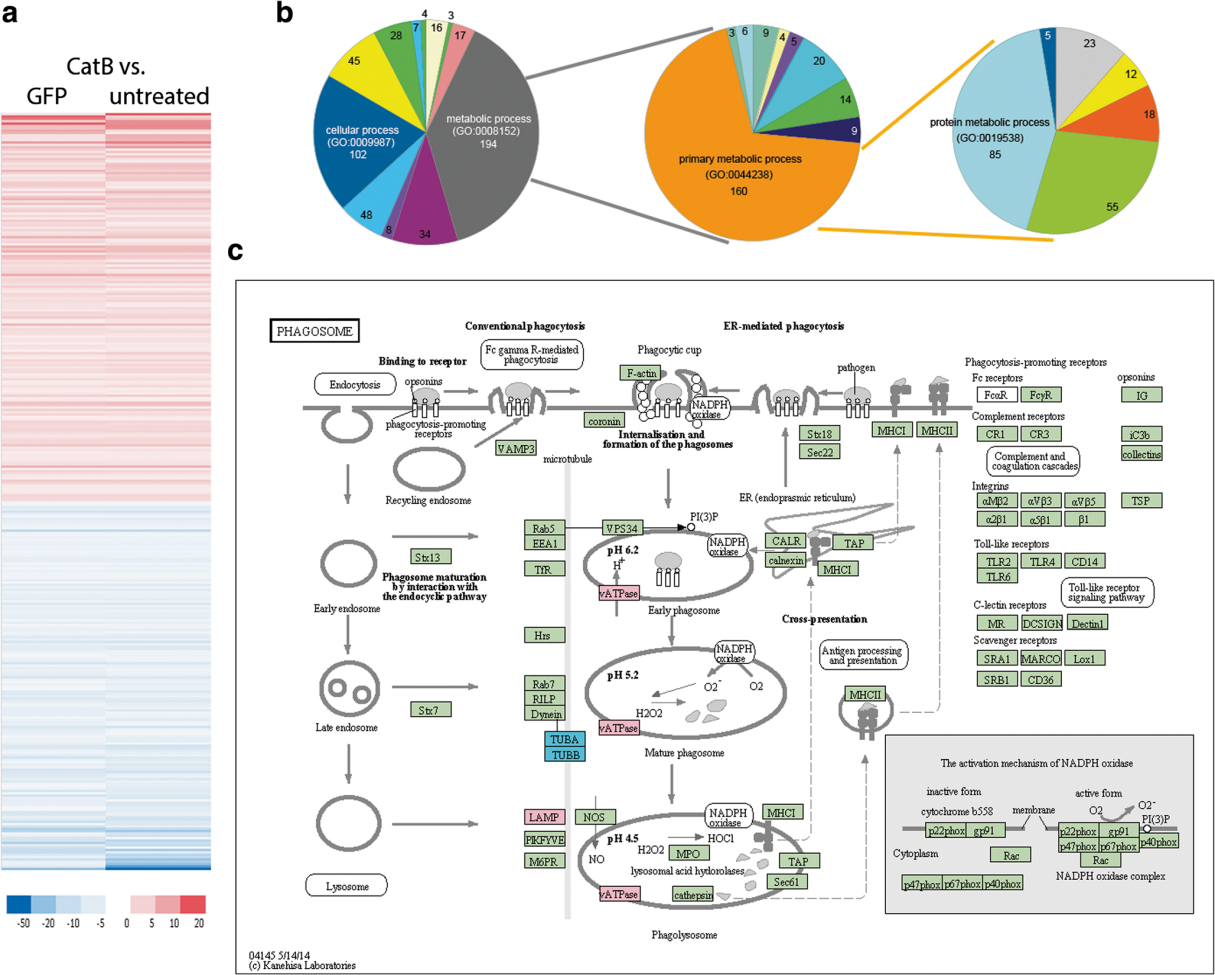
Proteomics changes induced by AdCatB. **a** Heatmap of 320 proteins that were significantly altered with AdCatB administration, common to both the AdGFP and untreated controls, indicating a near even amount of up- (164 proteins) and down- (156 proteins) regulation. **b** PANTHER analysis revealed enrichment of proteins involved in metabolic process. Expansion of this family of proteins identified primary metabolic processes as the major influencing subgroup, and a further expansion yielded protein metabolic processes as a primary enriched metabolic function with AdCatB treatment. **c** KEGG pathway analysis revealed that LAMP1 and vATPase, involved in the phagosomal compartments were significantly altered with AdCatB treatment as compared to AdGFP treated and untreated controls

The molecular mechanisms underlying CatB-mediated Aß degradation are unclear yet. To this end, we applied quantitative SWATH-MS proteomics to uncover proteins affected by CatB overexpression in NPC- neurons (Supplementary Data S1A). Upon comparison with AdGFP-infected control and with uninfected control, overall quantitative profiling identified 543 proteins that were significantly changed with CatB overexpression, as assessed by paired-samples z-scores (Supplementary Data S1B). Up- and down-regulated proteins in AdCatB-infected neurons were 49.4 and 50.6% of total (*n* = 268 and 275, respectively). A total of 320 out of 543 proteins were altered with 164 up- and 156 down-regulated in AdCatB treated cells in comparison with both AdGFP-infected and uninfected controls (Fig. 2a, Supplementary Data S1C). The biological functions of the identified proteins were assessed by PANTHER (Fig. 2b, Supplementary Data S1D). This was done as a multifaceted bioinformatic approach focused on analyzing altered biological processes, demonstrating that CatB overexpression had effects on a number of intracellular pathways, particularly those related to endolysosomal, phagosomal, and mitochondrial functions. Protein enrichment engaged in specific metabolic processes. Expansion of this family of proteins identified primary metabolic processes as a major altered cellular function (Fig. 2b, Supplementary Data S1E). Further investigation of these pathways utilizing the KEGG database showed that proteins within endolysosomal compartments such as Lamp1 and vATPase were up-regulated, indicating that CatB overexpression is involved in phagosome neuronal networks (Fig. 2c).

### AAV-Mediated CatB Expression

**Fig. 3:**
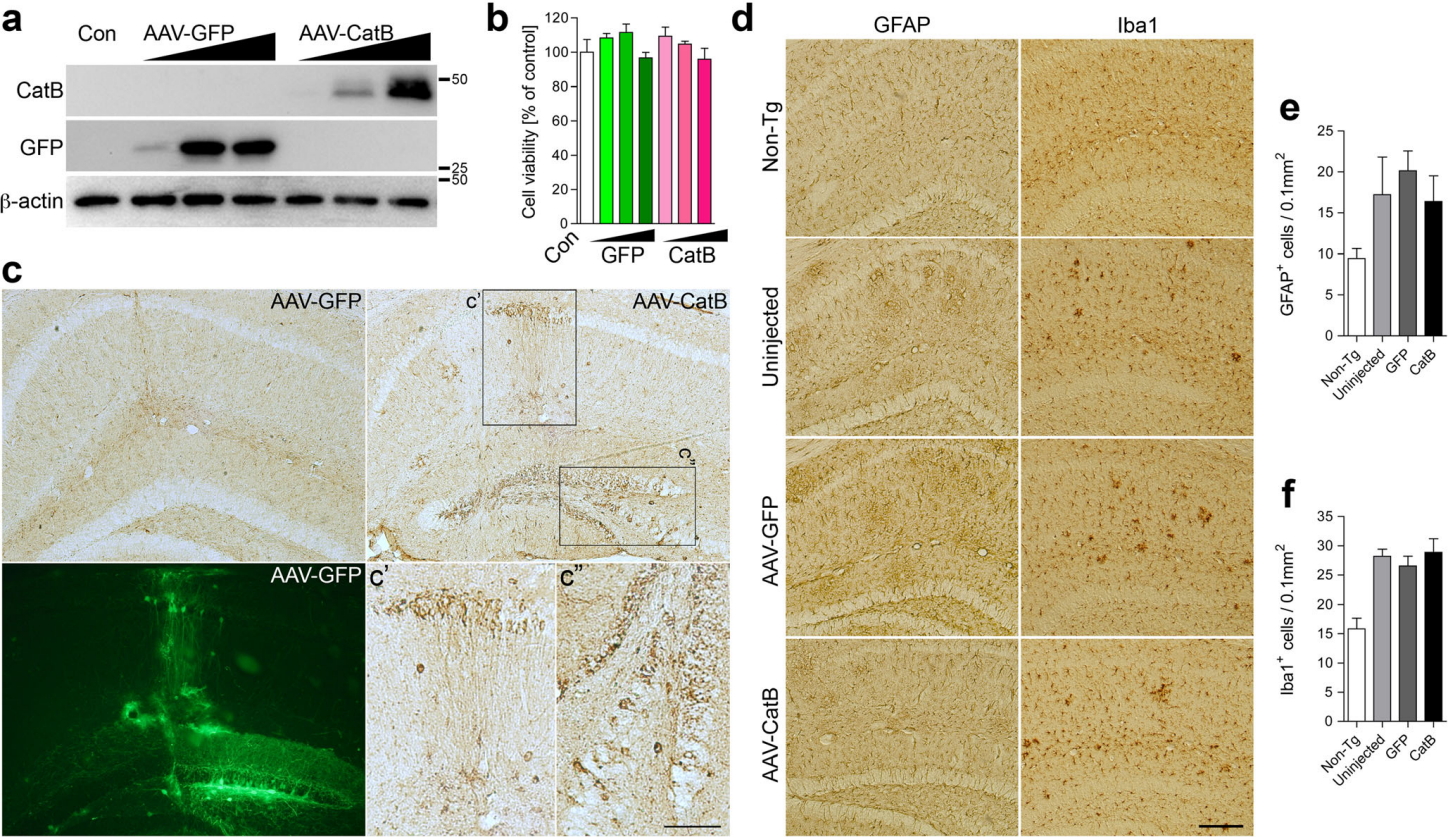
Optimization for AAV-GFP and CatB in vitro. **a** Immunoblots show expression of GFP and CatB in a dose-dependent manner in NPC-derived neurons transduced with AAVs at 1 × 10^7–9^ vg/10,000 cells/well. **b** Overexpression of CatB in NPC-derived neurons has no effect on cell viability as compared to control (Con) or GFP group. **c** Hippocampal frozen sections were immunostained for HA to identify exogenous CatB expression. HA-immunostaining and GFP fluorescent images in the AAV-GFP or CatB-injected hippocampus were shown. Scale bar =100 μm (50 μm in high magnified images). **d** Hippocampal frozen sections were immunostained for GFAP (astrocyte) or Iba1 (microglia). Scale bar =200 μm. **e** Quantification of GFAP-positive cells in the hippocampus. **f** Quantification of Iba-positive cells in the hippocampus

To assess CatB over-expression in an AD mouse model, AAV-CatB was generated. To examine AAV-CatB efficacy, NPC-derived neurons were transduced with AAV-GFP and AAV-CatB. Immunoblot analyses showed that the differential transduction of GFP and CatB were subsequently increased in a dose-dependent manner (Fig. 3a). To assess CatB neuronal effects, cell viability was measured using the MTT assay. AAV transduction of CatB did not alter cell viability when compared to control GFP groups (Fig. 3b). These results demonstrated high-level expression of CatB without neurotoxicity.

### CatB Attenuates Hippocampal Aß Levels in APP/PS1 Mice

**Fig. 4:**
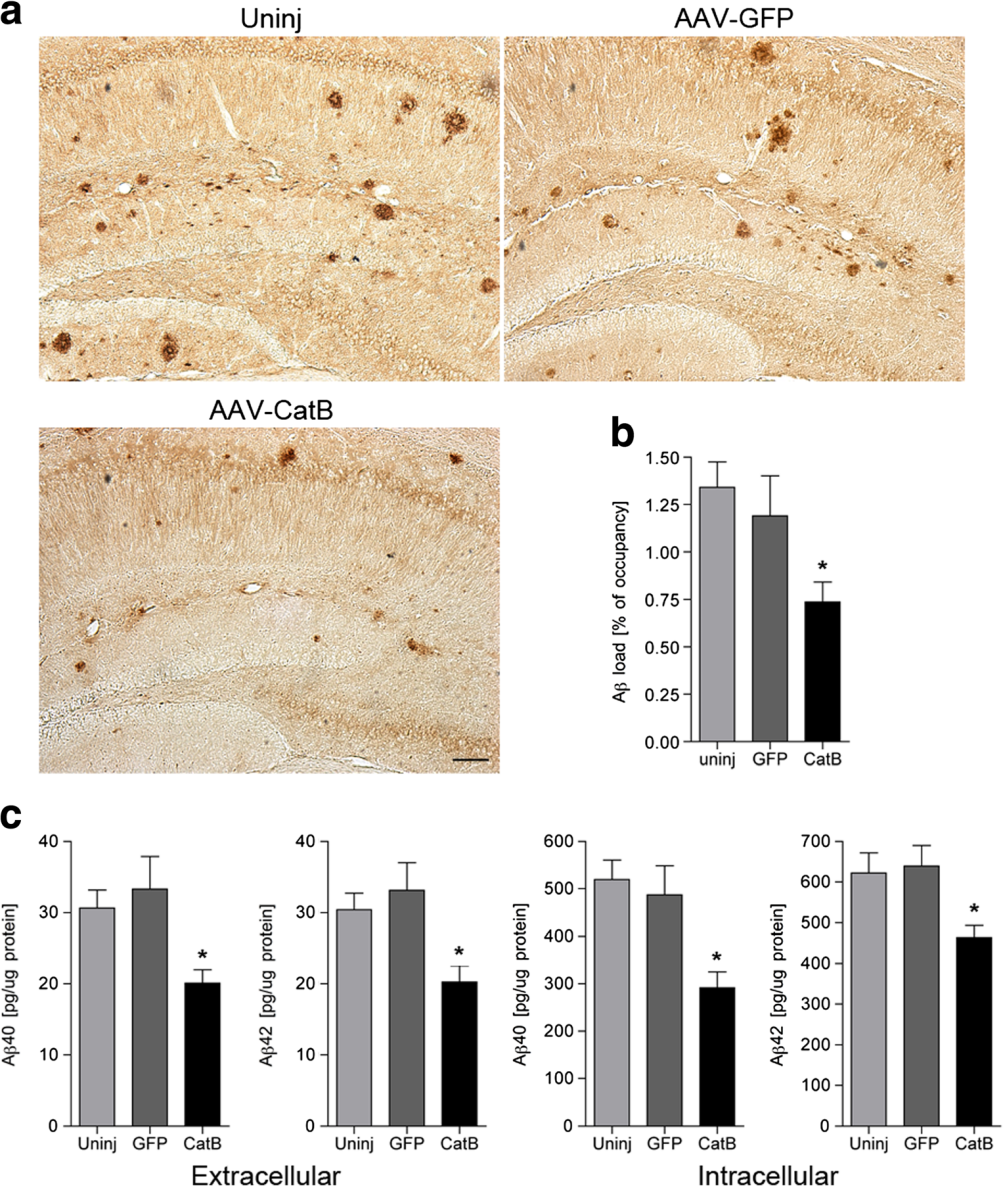
CatB attenuates Aß loads in the hippocampus of APP/PS1 mice. **a** Representative images of Aß staining in the hippocampus of AAV-GFP and AAV-CatB-injected APP/PS1 mice at 7 months of age. Scale bar =200 μm. **b** Quantification of total Aß loads in the hippocampal region (*n* = 7 per group, 12 sections per brain). **c** The levels of Aß40 and Aß42 in extracellular and intracellular-enriched fractions were measured by human Aß40 or Aß42-specific ELISA (*n* = 5). Bars represent mean ± S.E.M. *p < 0.05, one-way ANOVA, Newman-Keuls post hoc test

To elucidate the effect of CatB on ß-amyloidosis we injected AAV-GFP or -CatB (1 × 10^9^ vg/2 μl/shot) bilaterally into the hippocampus of three month-old APP/PS1 mice. Following animal sacrifice at seven months of age brains were secured. GFP and CatB expression in the hippocampus were confirmed at the terminal time point (Fig. 3c). Fluorescent analyses showed that efficient GFP expression was observed in the hippocampus injected with AAV-GFP, notably in pyramidal neurons and neuropils in Cornet d’Ammon (CA) 1 and the DG of the hippocampus. HA-immune-positive cells expressing exogenous CatB were observed in the AAV-CatB injected hippocampus, but not in AAV-GFP. At the time point expression of GFAP (astrocyte marker) or IbaI (microglial marker) is unchanged between the groups in APP/PS1 mice, demonstrating that CatB-overexpression does not induce astro/microgliosis (Fig. 3d-f). Neuropathological analyses for Aß (Fig. 4a) demonstrated that the AAV-CatB-injected group showed significant reductions in hippocampal Aß loads (23.2% reduction when compared to AAV-GFP group, Fig. 4b). To quantify the levels of Aß40 and Aß42, hippocampal homogenates were processed to separate extracellular-enriched fraction (extracellular Aß plaques) and intracellular-enriched fraction. Aß ELISA showed decreased Aß40 and Aß42 levels in both (extracellular Aß – 39.6% and 38.8% reduction for Aß40 and Aß42, intracellular levels – 40.2% and 27.4% reduction for Aß40 and Aß42, respectively when compared to AAV-GFP group, Fig. 4c). These data suggest that CatB attenuates ß-amyloidosis in the hippocampus.

### CatB Enhances Lamp1 Expression in Brain Tissues and in Neurons

**Fig. 5:**
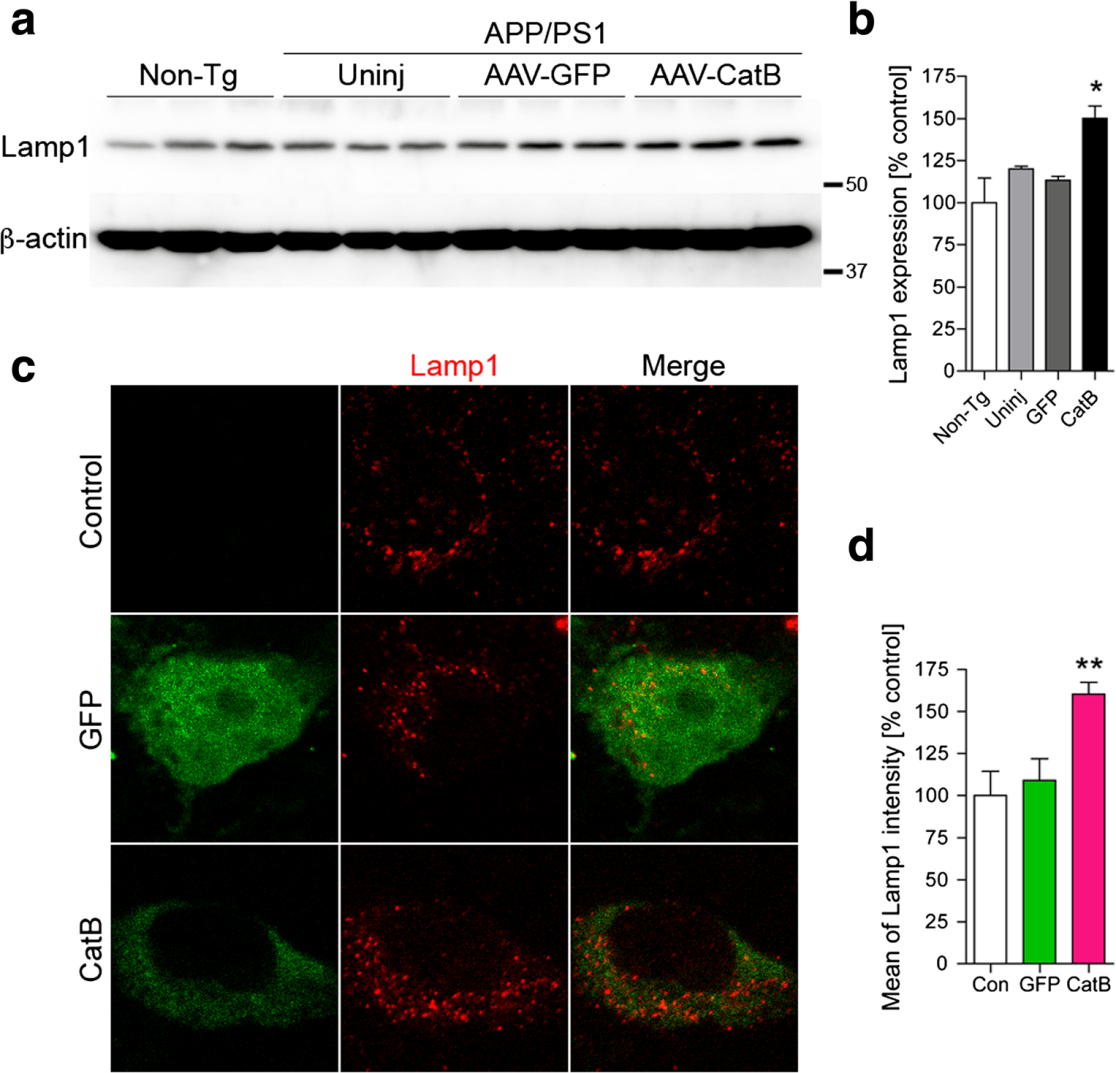
AAV-mediated CatB transduction increases Lamp1 expression in the hippocampus of APP/PS1 mice and cultured neurons. **a** Immunoblot of Lamp1 in intracellular fraction of the mouse hippocampus. **b** Quantification of Lamp1 expression (*n* = 6). **c** Confocal microscopy shows cellular localization of Lamp1 lysosomal compartment (red) in neurons. **d** Lamp1 expression levels were quantified using ImageJ (*n* = 3). Bars represent mean ± S.E.M. *p < 0.05, **p < 0.01, one-way ANOVA, Newman-Keuls post hoc test

To investigate the proteome of cells transduced by AAV-CatB intracellular fractions of hippocampal proteins were subjected to immunoblot assays (Fig. 5a). CatB overexpression increased Lamp1 levels by 68.0% when compared to the AAV- GFP-injected mouse group (Fig. 5b). To validate the results in cultured neurons, AAV-transduced cells were immunostained with an antibody to Lamp1 (Fig. 5c). Quantification on confocal microscopical images demonstrated that Lamp1 expression was enhanced in soma of neurons treated with AAV-CatB (60.3% increase to control, Fig. 5d). These data indicated that CatB induces the lysosomal activity.

### CatB Restores Spatial Learning and Memory

**Fig. 6:**
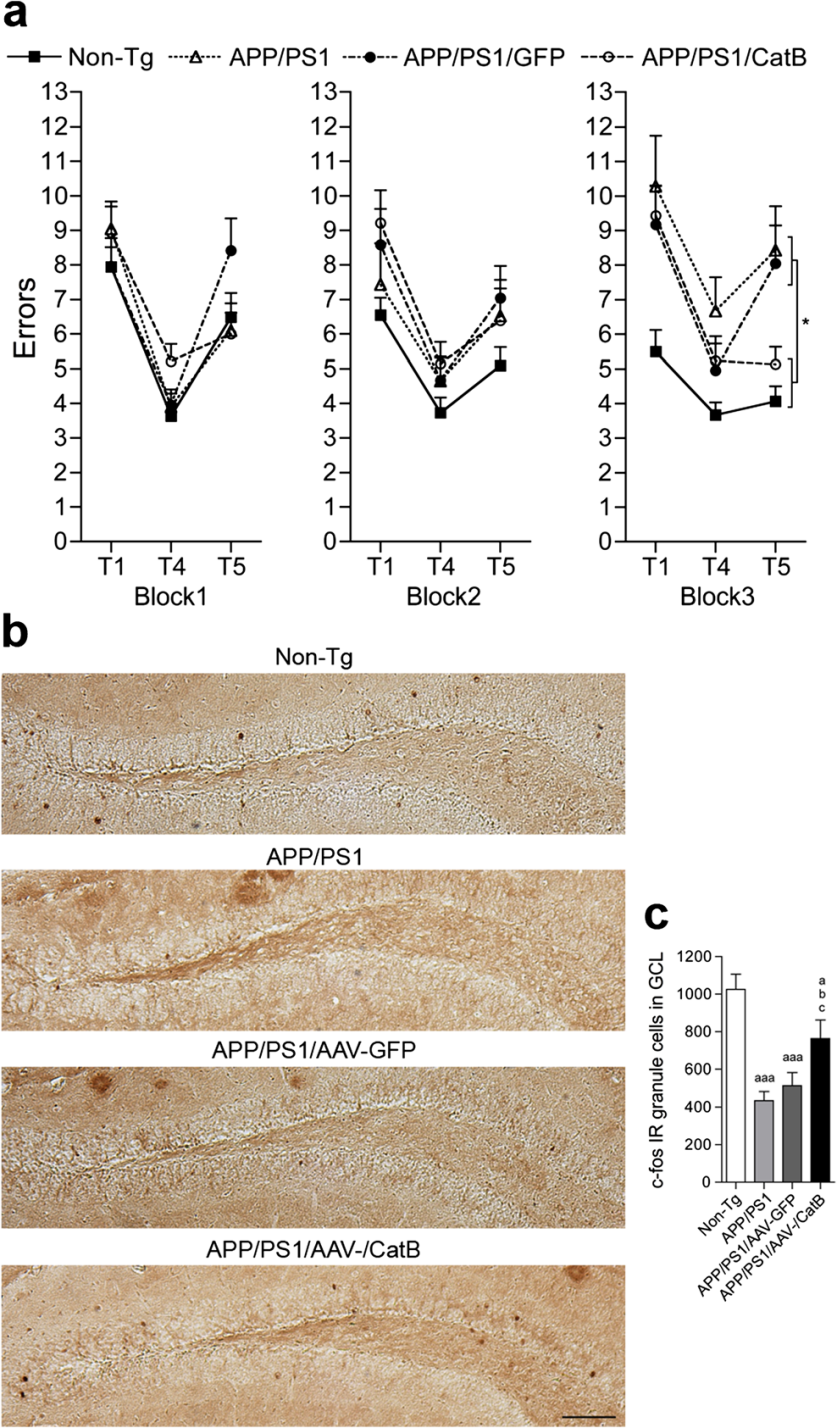
AAV-CatB-mediated transduction improves learning and memory in APP/PS1 mice. **a** Non-Tg (*n* = 10), AD (*n* = 9), AAV-GFP (*n* = 8) or CatB (*n* = 10)-injected APP/PS1 mice were tested by the RAWM task at 6–7 months of age. Non-Tg serves as a positive control for the spatial learning task. The compiled average errors for day 1–3, 4–6 and 7–9 are shown. Bars represent mean ± S.E.M. *p < 0.05, two-way ANOVA, Bonferroni post hoc test. **b** Immunohistochemical detection of c-fos-labeled cells in the dentate GCL. Scale bar, 200 μm. **c** Quantification of the number of c-fos-labeled cells (*n* = 7 mice per group, 12 sections per mouse). Bars represent mean ± SEM. ^a,b,c^ *p* < 0.05, ^aaa^ *p* < 0.001, ^a,aaa^ vs non-Tg, ^b^ vs uninjected APP/PS1, ^c^ vs AAV-GFP-injected APP/PS1, one-way ANOVA, Newman-Keuls post hoc test

To assess if AAV-CatB transduction effects learning and memory, we employed a RAWM task to assess memory acquisition and retention. These tests were performed in non-Tg, APP/PS1, and AAV-injected APP/PS1 mice. Three 3-day blocks for trial 1 (T1; randomized initial trial), T4 (final acquisition trial), and T5 (delayed retention trial) were used to evaluate the memory function at 6–7 months of age (Fig. 6a) (Kiyota et al. 2013). All animal groups showed reduced error numbers by T4 through three blocks. While the average number in non-Tg was lower than that in others at the third block, AAV-CatB-injected APP/PS1 mice showed lower number of errors with significant differences as compared to uninjected or AAV-GFP-injected APP/PS1 mice by T5. These results support our prior findings that APP/PS1 mice show impaired hippocampal function, memory acquisition and retention. The changes occurs by 6–7 months of age and were shown previously by RAWM tests (Diamond et al. 1999; Arendash et al. 2001; Kiyota et al. 2011; Kiyota et al. 2013). Improvements in APP/PS1 animals injected with AAV-CatB showed lower number of functional errors.

The proto-oncogene *c-fos* is one of the immediate-early genes that are induced by neural activity and behavior, and known to play a role in the neuroplastic mechanisms for memory consolidation (Palop et al. 2003; Kiyota et al. 2011). To address if memory improvement in AAV-CatB-injected APP/PS1 mice were associated with c-fos expression, the numbers of c-fos-immunoreactive (c-fos^+^) neurons were evaluated in the granular cell layer (GCL) of the DG (Fig. 6b). While the number of c-fos^+^ neurons in both uninjected and AAV-GFP-injected APP/PS1 mice was reduced compared to nonTg control (57.7% and 49.8% reduction, respectively), the numbers in AAV-CatB-injected APP/PS1 mice were similar to that in non-Tg controls (56.9% and 67.4% increase of uninjected and AAV-GFP-injected APP/PS1 mice, respectively) (Fig. 6c). These data suggest that CatB treatment can recover learning and memory functions.

## Discussion

Aß induces neuroinflammation with consequent synaptic and neuritic injury, and tau hyperphosphorylation. These processes eventually lead to neuronal death and memory impairment and are characteristic of the pathobiology of AD (Mattson 2004; Billings et al. 2005; Oakley et al. 2006). Based on these observations, therapeutic efforts have largely been made through targeting Aß. As seen over the past decade breakdown of toxic Aß accumulation remains a major target to ameliorate disease. Such targeting strategies have been focused on Aß removal by both active and passive Aß immunization. Notably, Aß clearance is associated with improved memory function in AD mouse models (Huang and Mucke 2012). Nonetheless, clinical trials employing such immunization strategies thus far have shown disappointing result. Untoward immunological responses and more limited improvement in clinical symptoms with worsened disease outcomes were reported following immunizations (Lemere 2013; Panza et al. 2014). This suggests that removing Aß, particularly extracellular deposits, may not yield improvements in disease outcomes. Hence the outcomes are likely due to continuous Aß production in the AD brains. Targeting lysosomal degradation pathways to affect APP/Aß trafficking, specifically leading to degradation and clearance of Aß within neurons is a likely promising approach to slow the pathological progression of human disease (Kiyota et al. 2015b).

As CatB functions in protein degradation during phagocytosis or autophagy (Alvarez et al. 2012) harnessing this protein as a therapeutic agent and notably by affecting LAMP1 regulation is a realistic mechanism forward towards developing a novel disease treatment strategy. In the human brain, CatB is expressed in glial and in endothelial cells of vascularised glioblastomas, and serves as a predictor of shorter survival in brain tumors (Levicar et al. 2003). In the current study overexpression of neuronal CatB did not affect cell viability. In addition, no changes in astrocyte or microglial responses were seen. This highlights that CatB-overexpression elicits no untoward effects due to neuron-specific transduction of AAV (Kiyota et al. 2009). As aforementioned abundant CatB is expressed in neuronal perikarya as well as extracellularly associated within senile plaques in the postmortem brain of AD patients (Cataldo et al. 1990), and lysosomal protease-mediated APP processing in degenerating neurons is considered to develop the plaques (Cataldo and Nixon 1990). Hence reduction in CatB expression or genetic deficiency of CatB decreases Aß levels and improved memory function in rodent models of AD (Hook et al. 2007; Hook et al. 2008; Hook et al. 2009; Hook et al. 2011; Kindy et al. 2012). On the one hand, overexpression of CatB also lowers Aß levels (Mueller-Steiner et al. 2006; Yang et al. 2011; Wang et al. 2012). Morevoer, we now show that cognitive function was improved by AAV-CatB treatments in our AD mouse model. This was shown by performance in the RAWM task and further confirmed by immunohistological analysis, suggesting a possible therapeutic approach for compensation of lower expression of CatB in AD patients with more profound dementia (Tiribuzi et al. 2014).

Treatment with CatB lead to a variety of changes in the neuronal proteome with particular focus on cell-associated phagosomal and mitochondrial pathways. Dysregulation of the endolysosomal and autophagosomal pathways is a significant contributing factor in the aggregation of Aß, possibly by both contributing to the genesis of toxic, aggregation-prone Aß subspecies and by lack of degradation and ultimately causing lysosomal leakage. Lysosomal function is tied closely with its acidic pH, with an optimal pH of 4.5–5.0. Such acidification is tightly regulated by the lysosomal membrane and its proteins, including LAMP1 and V-ATPase (Shen and Mizushima 2014). Both, interestingly, were found to be significantly upregulated in CatB-expressed neurons. The lysosomal V-ATPase complex senses contents of the lysosomal and controls the acidification of the lumen, contributing to degradation. This suggests the mechanism behind the degradation of Aß, as shown by a general decrease in Aß40 and Aß42 levels in ELISA analysis, is directly related to lysosomal function.

## Conclusion

Overall, we now demonstrate that recombinant CatB overexpression results in reduced Aß production in both cultured neurons and AD mouse model brains. AAV-CatB injection ameliorates AD pathobiology including ß-amyloidosis and impairments in learning and memory in the mouse brain. These effects are caused by promoting lysosomal degradation, specifically related to Aß metabolism. Taken together, these findings support the idea that CatB has therapeutic potential for ameliorating the signs and symptoms of AD.

## Electronic supplementary material

ESM 1 (XLSX 247 kb)

## Abbreviations

Aß: Amyloid-ß
APP: Aß precursor protein
AD: Alzheimer’s disease
CatB: Cathepsin B
Ad: Adenovirus
NPC: Neural progenitor cell
AAV: Adeno-associated virus
PS1: Presenilin-1
Tg: Transgenic
GFP: Green fluorescent protein
CysB: Cystatin B
SWATH-MS: Sequential window acquisition of all theoretical fragment ion spectra - mass spectrometry
PANTHER: Protein Analysis Through Evolutionary Relationships
KEGG: Kyoto Encyclopedia of Genes and Genomes
MTT: 3-(4,5-dimethylthiazol-2-yl)-2,5-diphenyltetrazolium bromide
GFAP: Glial fibrillary acidic protein
Iba1: Ionized calcium binding adaptor molecule 1
Lamp1: Lysosome-associated membrane protein-1
GCL: Granular cell layer
DG: Dentate gyrus
RAWM: Radial arm water maze
